# Avenanthramide A triggers potent ROS-mediated anti-tumor effects in colorectal cancer by directly targeting DDX3

**DOI:** 10.1038/s41419-019-1825-5

**Published:** 2019-08-07

**Authors:** Rong Fu, Peng Yang, Zongwei Li, Wen Liu, Sajid Amin, Zhuoyu Li

**Affiliations:** 1 Institute of Biotechnology, Key Laboratory of Chemical Biology and Molecular Engineering of National Ministry of Education, Shanxi University, Taiyuan, 030006 China; 2 Institutes of Biomedical Sciences, Shanxi University, Taiyuan, 030006 China; 3 Department of Lymphoma and Myeloma, Center for Cancer Immunology Research, The University of Texas MD Anderson Cancer Center, Houston, Texas 77030 USA; 4 School of Life Science, Shanxi University, Taiyuan, 030006 China

**Keywords:** Cancer prevention, Target validation

## Abstract

Colorectal cancer (CRC) is a common malignant gastrointestinal tumor with high mortality worldwide. Drug resistance and cytotoxicity to normal cells are the main causes of chemotherapeutic treatment failure in CRC. Therefore, extracting the bioactive compounds from natural products with anti-carcinogenic activity and minimal side-effects is a promising strategy against CRC. The present study aims to evaluate the anti-carcinogenic properties of avenanthramides (AVNs) extracted from oats bran and clarify the underlying molecular mechanisms. We demonstrated that AVNs treatment suppressed mitochondrial bioenergetic generation, resulting in mitochondrial swelling and increased reactive oxygen species (ROS) production. Further study indicated that AVNs treatment significantly reduced DDX3 expression, an oncogenic RNA helicase highly expressed in human CRC tissues. DDX3 overexpression reversed the ROS-mediated CRC apoptosis induced by AVNs. Of note, we identified Avenanthramide A (AVN A) as the effective ingredient in AVNs extracts. AVN A blocked the ATPase activity of DDX3 and induced its degradation by directly binding to the Arg287 and Arg294 residues in DDX3. In conclusion, these innovative findings highlight that AVNs extracts, in particular its bioactive compound AVN A may crack the current hurdles in the way of CRC treatment.

## Introduction

Colorectal cancer (CRC) is one of the most common tumors of the digestive tract; with high morbidity and mortality rates worldwide^1^. Due to obtrusive drug resistance and potent side effects, the outcomes of current chemotherapy drugs are not so desirable^2^. Therefore, exploring the bioactive anti-cancer compounds derived from natural products with low toxicity and minimal side effects has emerged as a hot topic in cancer biology.

Oats (*Avena sativa L*.) is a wholegrain cereal and an important edible crop, which contains several bioactive ingredients^3^. Epidemiological studies have reported that the long term consumption of oats positively affect the digestive health and minimize the risk of CRC^4^. Oats bran, a familiar byproduct of refined oats milling, has attracted considerable attention as they contain many bioactive products including β-glucan, vitamins, and avenanthramides (AVNs). Pharmacological studies have shown that AVNs, which belong to the phenolic alkaloids and are unique to oats, exhibit anti-inflammatory, antioxidative, and anti-cancer properties^5,6^. However, the specific compounds responsible for anti-carcinogenic characteristics of AVNs remain unclear. Therefore, it is urgent to identify and characterize the effective anti-carcinogenic agents of AVNs and to figure out the underlying molecular mechanisms.

The imbalance between free radical generation and intracellular scavenging activity give rise to the production of excess level of reactive oxygen species (ROS). Mitochondrial ROS generation is largely coupled to mitochondrial respiration. Electron leakage at the electron transport chain (ETC) complex I and III leads to partial reduction of oxygen to form superoxide. Excessive ROS production could activate the permeability transition pore (PTP) and leads to the loss of mitochondrial membrane potential (MMP), resulting in the release of cytochrome c, caspase activation and apoptosis initiation^7^. Unlike normal cells, tumor cells generate a massive quantity of ROS to maintain their malignant phenotype, which makes them more vulnerable to oxidative stress. Further elevation of ROS levels to exceed the cellular tolerability threshold, will lead to cancer cell death^8^. DDX3, a multifunctional RNA helicase, is often highly expressed in several tumor types to promote cancer progression^9^. DDX3 has been documented to facilitate tumor invasiveness and cetuximab resistance in KRAS wild-type colorectal cancer^10–12^. Recent studies have shown that DDX3 suppression leads to mitochondrial collapse and increases the ROS levels via affecting the mitochondrial translation. Thus, DDX3 may be a novel effective strategy for CRC trial.

This study aims to investigate the anti-tumor effects and underlying molecular mechanisms of AVNs extracted from oats bran. Our results demonstrate that AVNs induce mitochondrial bioenergetics collapse and ROS-dependent apoptosis in CRC cells. We further identified Avenanthramide A (AVN A) as the main active compound in AVNs extract, which binds to DDX3 protein at Arg287 and Arg294 residues to inhibit its ATP hydrolysis activity and protein stability. Taken together, our study highlights that AVN A is a promising bioactive molecule that can be exploited in therapeutic practices against CRC.

## Results

### AVNs attenuate cell growth and viability in colon cancer cells

**Fig. 1:**
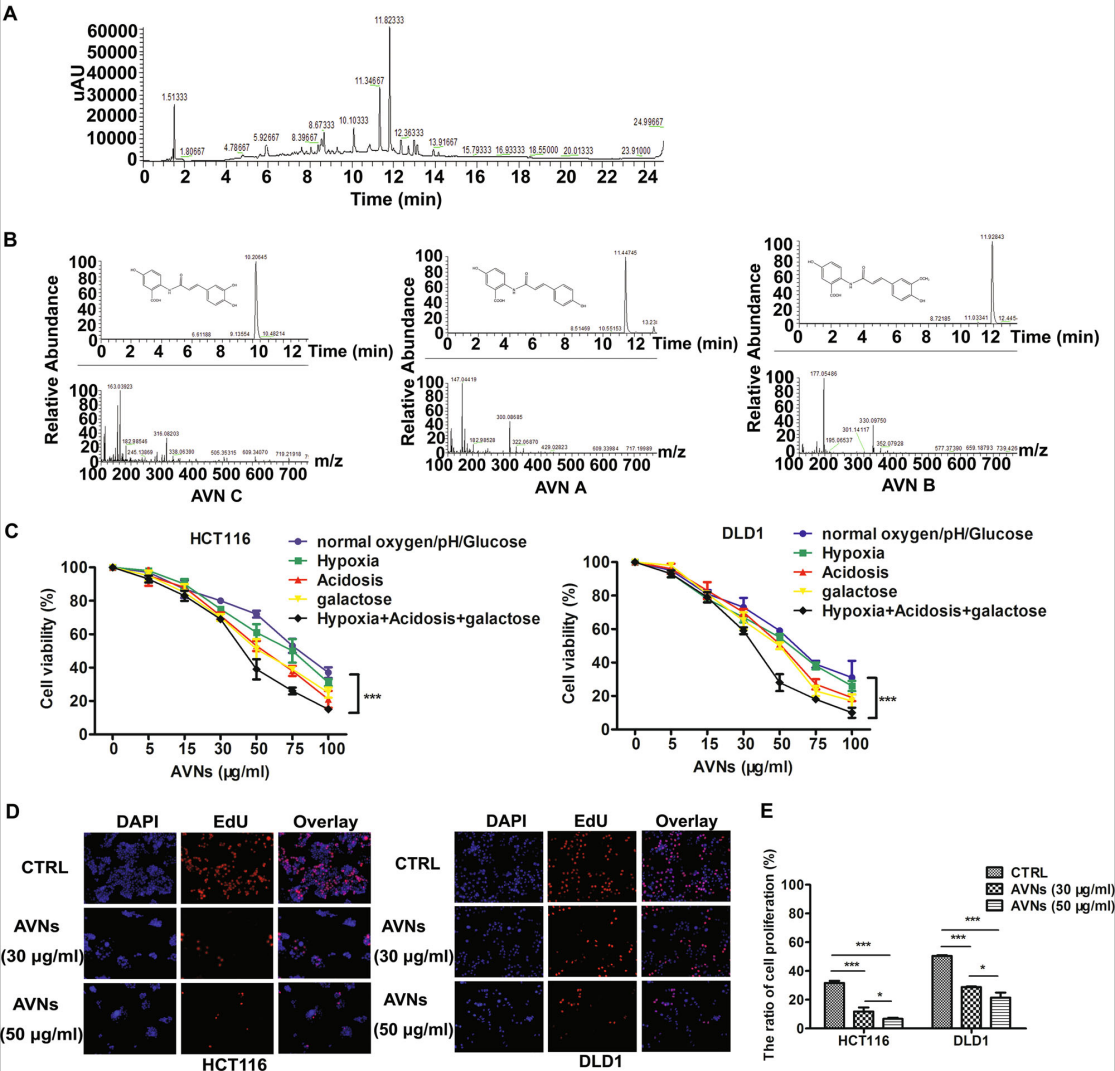
AVNs extracted from oats bran inhibited growth and viability of CRC cells. **a** HPLC chromatograms at 280 nm of oats bran extracts. **b** The MS spectra of peak avenanthramide A, avenanthramide B, avenanthramide C in oats bran extracts were obtained on ion-trap MS. **c** Dose-dependent activity of AVNs in HCT116 and DLD1 on indicated conditions as assessed by MTT assay. (*n* = 3, mean ± SD). **d** EdU assay showed proliferation rate of HCT116 and DLD1 cells treated with AVNs. **e** Columns showed mean values of three experiments and expressed as mean ± SD. *P < 0.05, **P < 0.01, ***P < 0.001. *P*-values were calculated by a Student’s *t*-test. All experiments have been replicated three independent times

Before investigating the anti-tumor effects of AVNs, the HPLC-MS was performed to detect and tentatively identify the phytochemical compounds in oats bran extracts. Figure 1a showed the representative HPLC chromatograms of oats bran extracts. Three strong signal peaks around 10–12 min were selected and further confirmed by mass spectrometry. Identification was determined on the basis of protonated molecular ions [M + H]^+^, according to Hitayezu et al., signals with *m/z* values of 300, 330 and 316 were considered as avenanthramide A (AVN A), avenanthramide B (AVN B), and avenanthramide C (AVN C), respectively (Fig. 1b)^13^. Next, to evaluate the anti-tumor effects of AVNs, CRC cells (DLD1, HCT116, SW480, SW620) and normal colonic epithelial cells (FHC) were treated with AVNs. AVNs treatment resulted in a significant loss of viability in CRC cells in a time-dependent manner, especially in DLD1 and HCT116 cells; whereas AVNs exhibited negligible effect on viability of FHC cells (Supplementary Fig. S1A). Solid tumors usually grow under abnormal pathophysiological conditions such as glucose starvation, hypoxia and acidic extracellular pH^14^. Interestingly, the anti-tumor capacities of AVNs were more prominent in the above-mentioned stress conditions than in normal culture conditions (Fig. 1c). EdU assay showed that AVNs treatment significantly attenuated CRC cell proliferation (Fig. 1d, e). Consistently, AVNs also suppressed the clonal formation ability of CRC cells in a dose-dependent manner (Supplementary Fig. S1B, C).

### AVNs induce mitochondrial dysfunction in colon cancer cells

**Fig. 2:**
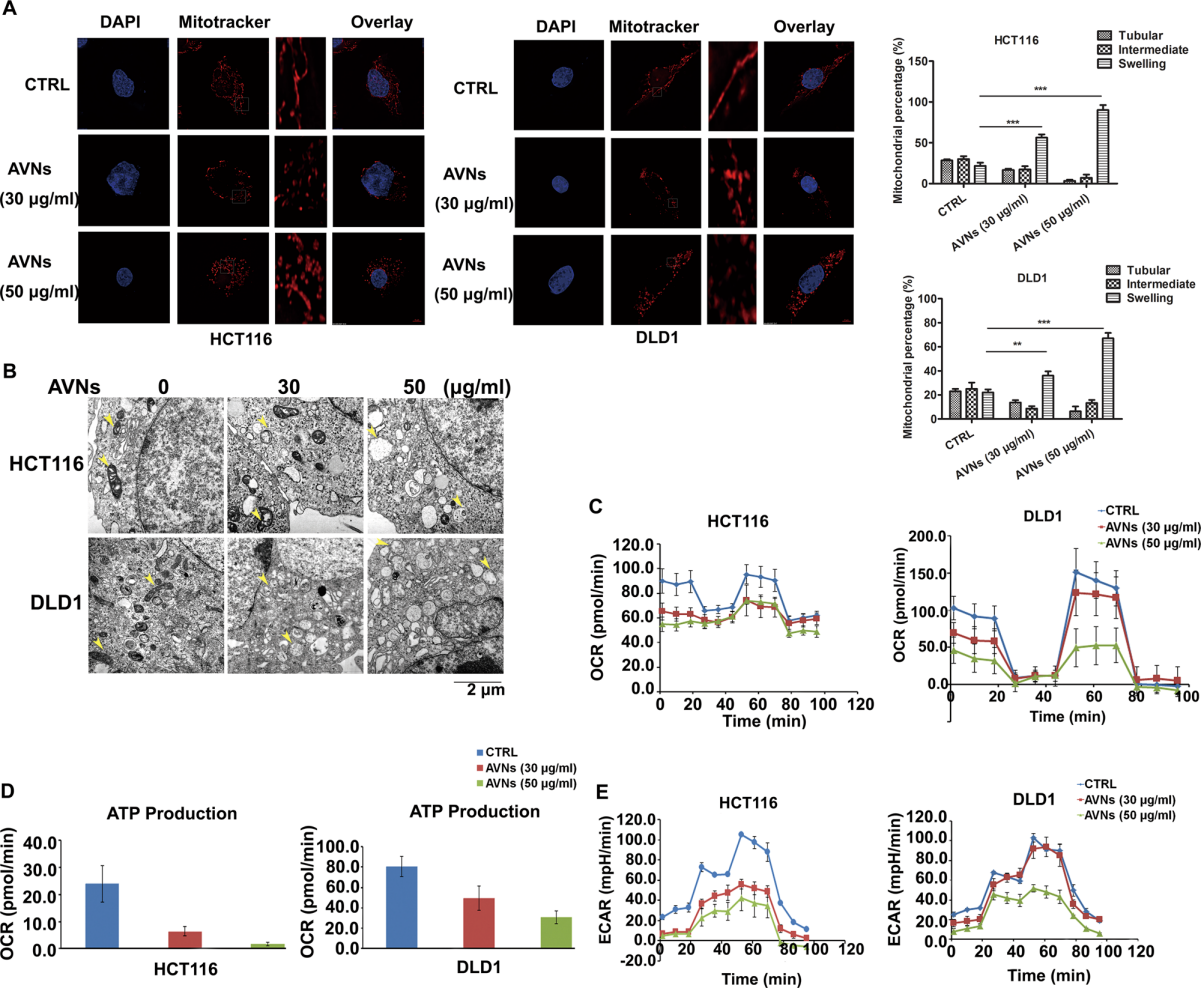
AVNs-induced mitochondrial dysfunction in CRC cells. **a** Representative images of mitochondrial morphology in CRC cells treated with AVNs for 24 h visualized by labeled with MitoTracker Red. Scale bars represent 6 μm (left panel). Quantification of mitochondrial morphology in CRC cells (right panel). **b** Representative TEM images of HCT116 and DLD1 cells fixed after treated with AVNs for 24 h. Arrowheads represents the respective Mitochondria. Scale bars represent 2 μm. **c** Oxygen consumption rate (OCR) of HCT116 and DLD1 cells were measured in the presence of AVNs for 24 h (*n* = 3). **d** ATP production was determined in both HCT116 and DLD1 cells with the indicated concentrations of AVNs treatment. **e** HCT116 and DLD1 cells treated with the indicated concentrations of AVNs for 24 h, and ECAR was measured after consecutive injections of glucose (10 mM), oligomycin (1 μM), and 2-DG (50 mM) (n = 3). *P < 0.05, **P < 0.01, ***P < 0.001. *P*-values were calculated by a Student’s *t*-test. All experiments have been replicated three independent times

Mitochondrion is the primary endogenous source for ROS generation. AVNs have been shown to possess antioxidant activities in vitro. We wonder whether AVNs exert anti-tumor properties through impinging on mitochondrial function. To this end, we used MitoTracker Red, a fluorescent mitochondrial dye, to monitor mitochondrial morphology. As shown in Fig. 2a, the vehicle control group showed a balanced proportion of tubular, intermediate and fragmented morphology, while the swelling and fragmentation types were increased after AVNs treatment (Fig. 2a). Transmission electron microscopy (TEM) results further confirmed the mitochondrial swelling in DLD1 and HCT116 cells after AVNs treatment (Fig. 2b). To determine the possible effect of AVNs on mitochondrial function and cellular bioenergetics, the cellular oxygen consumption rate (OCR) of mitochondrial respiration in CRC cells was measured. AVNs treatment clearly decreased the basal respiration, proton leak, spare respiratory capacity and maximal respiration levels in CRC cells (Fig. 2c). Similarly, ATP generation was significantly attenuated after 24 h of AVNs treatment (Fig. 2d). Some agents that inhibit ATP production by oxidative phosphorylation may produce a compensatory increase in glycolysis, characterized by increased extracellular acidification rate (ECAR)^15^. Thus, we monitored the ECAR of DLD1 and HCT116 cells in the absence and presence of AVNs. Interestingly, AVNs did not caused a significant increase in ECAR, and it appeared (at concentration of 50 ug/ml) to produce a noticeable reduction in the glycolysis, glycolytic capacity, and glycolytic reserve of CRC cells (Fig. 2e). All together, these results demonstrate that AVNs treatment triggers mitochondrial swelling and blocks mitochondrial bioenergetic generation, resulting in mitochondrial dysfunction.

### AVNs impair ETC complexes and induce ROS production

**Fig. 3:**
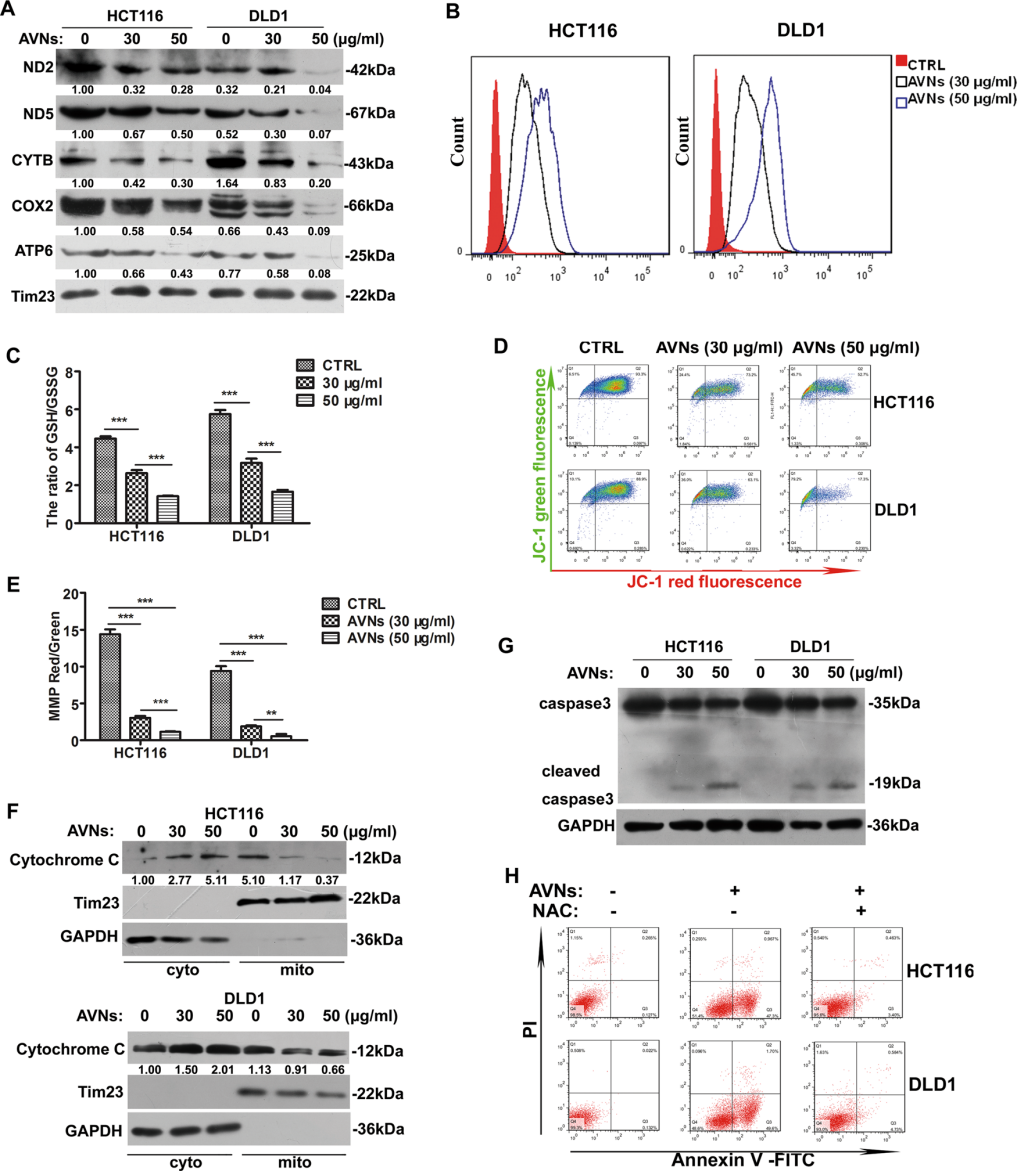
AVNs attenuated mitochondrial translation and increased ROS production to induce apoptosis of CRC cells. **a** Western blot showing ND2, ND5, CYTB, COX2, and ATP6 in AVNs-treated CRC cells. **b** Intracellular ROS levels in HCT116 and DLD1 treated with indicated concentrations of AVNs were measured by flow cytometry. **c** The GSH and GSSG were measured respectively, and the ratio of GSH/GSSG was calculated in HCT116 and DLD1 cells treated with AVNs for 24 h. (*n* = 3, mean ± SD). **d** JC-1 dye was used to detect effects of AVNs treatment on MMP levels of HCT116 and DLD1 cells by flow cytometry. **e** The MMP levels derived from (D) was indicated as the ratio of red/green fluorescence. **f** Western blot was used to measure the expressions of cytochrome c in HCT116 and DLD1 cells treated with indicated concentrations of AVNs. Tim23 and GAPDH served as mitochondrial marker and cytosolic marker respectively. **g** The expression of cleaved-caspase 3 in both HCT116 and DLD1 cells following the treatment with indicated concentrations of AVNs for 24 h was measured and normalized by GAPDH. **h** Flow cytometry analysis depicts the effects of AVNs on apoptotic CRC cells stained with Annexin V/PI staining. HCT116 and DLD1 cells were pretreated with 10 mM NAC for 2 h and cultured with AVNs for 24 h. *P < 0.05, **P < 0.01, ***P < 0.001. *P*-values were calculated by a Student’s *t*-test. All experiments have been replicated three independent times

We next sought to determine whether AVNs-induced mitochondrial dysfunction is associated with ETC complexes. As shown in Fig. 3a, AVNs treatment pointedly reduced the protein levels of ND2, ND5, CYTB, COX2, and ATP6, which are the subunits of of ETC complexes encode by mitochondrial genes (Fig. 3a). ETC complexes I and III are the main sources of intracellular ROS generation^16^. Disruption of ETC complex results in the leakage of electrons, which can be captured by oxygen to produce ROS^17^. Herein, we used ROS-sensitive fluorescent probe DCFH-DA to evaluate the effects of AVNs on intracellular ROS levels. Flow cytometry analysis demonstrated that AVNs treatment significantly induced ROS production in a concentration-dependent manner in CRC cells, but not in normal cells (Fig. 3b, Supplementary Fig. S1D). Consistently, the increased ROS production was parallel with decreased reduced glutathione/oxidized glutathione ratio (GSH/GSSG) (Fig. 3c). JC-1 staining further indicated that MMP was depolarized in response to AVNs treatment (Fig. 3d, e). AVNs treatment promoted the release of mitochondrial cytochrome c and activation of caspase-3 in CRC cells (Fig. 3f, g). Importantly, the apoptosis induced by AVNs could be blocked by a potent antioxidant N-acetylcysteine (NAC) (Fig. 3h), indicating AVNs-induced apoptosis in CRC cells through impairing ETC complexes and inducing oxidative stress.

### DDX3 expression in human colon cancer samples

**Fig. 4:**
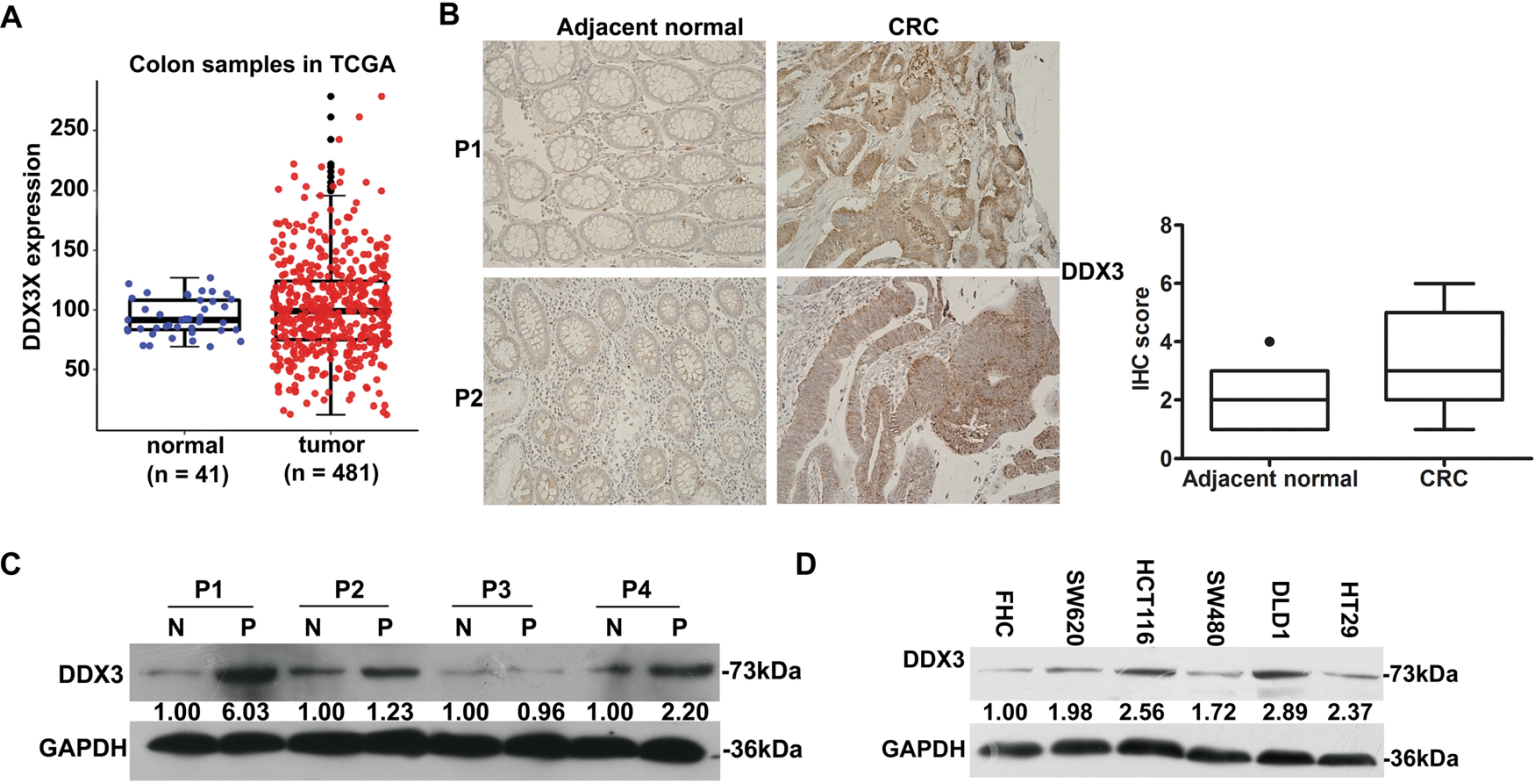
The expression of DDX3 is upregulated in CRC samples. **a** The TCGA analysis shown the differential of DDX3 expression between primary tumor (*n* = 481) and normal (*n* = 41). *P* = 0.00619. **b** Representative immunohistochemical staining showing DDX3 expression in CRC and adjacent normal colonic tissues. *n* = 53 for each group and IHC scoring was performed in both groups according to the staining intensity. **c** Western blotting depicts the DDX3 levels in CRC tissues and adjacent normal colonic tissues in four typical patients. **d** Western blot was used to determine the level of DDX3 expression in normal colonic epithelial cells and CRC cells

Growing number of studies have confirmed that DDX3, an RNA helicase with oncogenic properties, plays a vital role in mitochondria translation and tumor progression^18,19^. By analyzing The Cancer Genome Atlas (TCGA), we observed that DDX3 expression was much higher in CRC tissues than in normal tissues (Fig. 4a). Herein, we compared DDX3 expression between 53 matched pairs of CRC and adjacent normal tissues by IHC staining. As shown in Fig. 4b, the DDX3 expression in CRC tissues was significantly higher than that in adjacent normal tissues. These results were further confirmed in four pairs of fresh CRC and adjacent normal tissues by western blotting (Fig. 4c). Interestingly, the expression pattern of DDX3 in different CRC cell lines was highly correlated with their sensitivities to AVNs treatment. That is, cell lines (HCT116 and DLD1) with a higher DDX3 expression have a better response to AVNs treatment (Fig. 4d), suggesting that AVNs induce CRC cell apoptosis via targeting DDX3. In addition, compared with normal culture conditions, the protein levels of DDX3 were increased obviously under the stress milieus (combination of nutrient depletion, hypoxia, and low extracellular pH) (Supplementary Fig. S2A).

### AVNs target DDX3 to induce ROS-dependent apoptosis

**Fig. 5:**
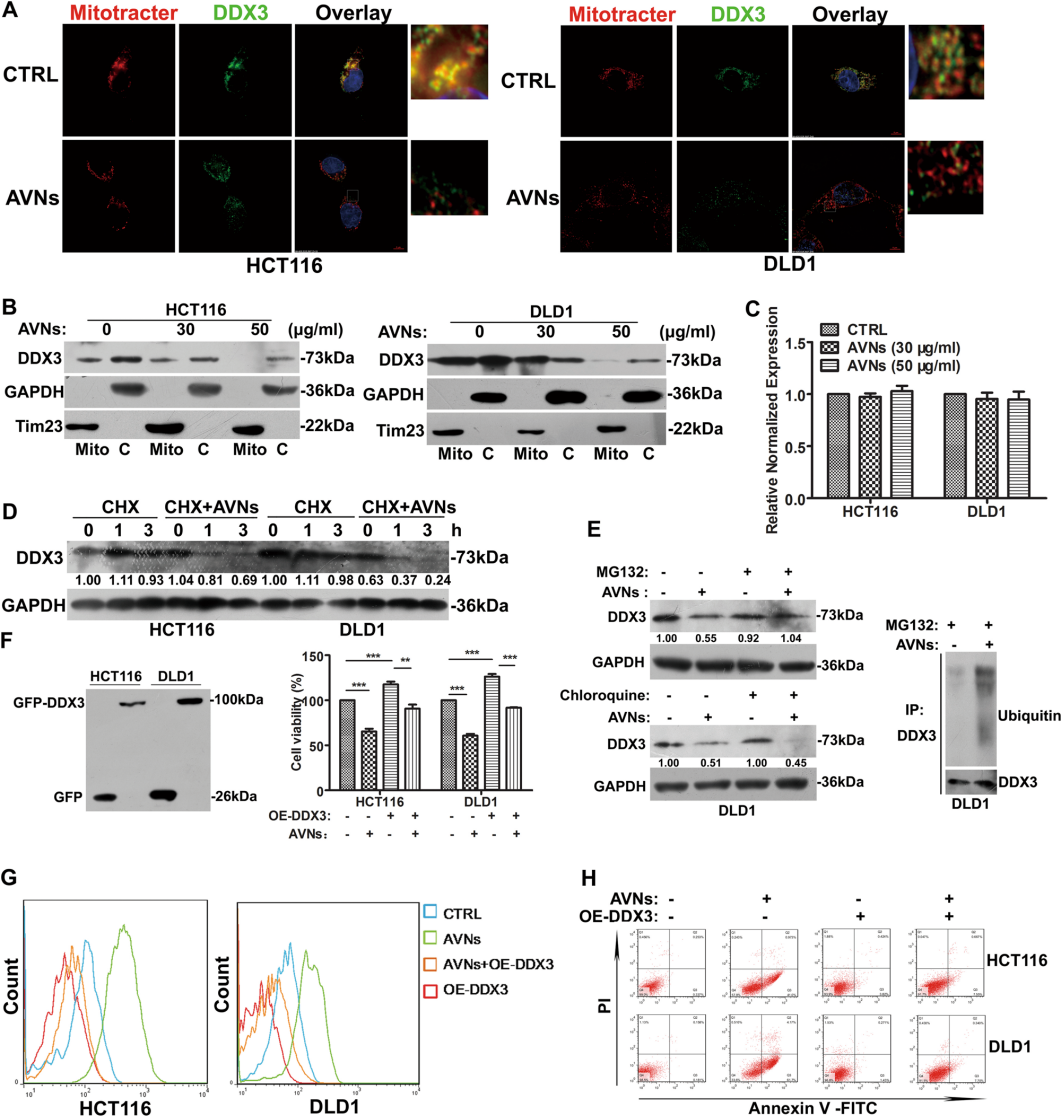
AVNs blocked mitochondrial translation via DDX3 to trigger apoptosis of CRC cells. **a** HCT116 and DLD1 cells treated with AVNs of 50 μg/ml for 24 h and stained with an anti-DDX3 antibody, MitoTracker, and DAPI. Scale bars represent 6 μm. **b** HCT116 and DLD1 cells treated with indicated concentration of AVNs and the expression of DDX3 was performed by western blot in cytosolic (C) and mitochondrial (mito) fractions of cell lysates. Tim23 and GAPDH served as mitochondrial marker and cytosolic marker respectively. **c** The effect of AVNs treatment on the mRNA expression levels of DDX3 was analyzed. (*n* = 3, mean ± SD). **d** HCT116 and DLD1 cells treated with the indicated concentrations of AVNs in the presence of 20 μM CHX for 0, 1, 3 h, respectively. DDX3 expression was detected by western blot. **e** DLD1 cells were treated with 50 μg/ml AVNs for 24 h with or without 20 μM MG132 or 30 μM chloroquine. The protein level of DDX3 was detected by western blot (Left panel). DLD1 cells were treated with AVNs for 24 h, followed by the addition of 20 μM MG132 for an additional 6 h. Cell extracts were subjected to immunoprecipitation with anti-DDX3 antibody. The conjugates were detected with anti-ubiquitin and anti-DDX3 antibody (Right panel). **f** The DDX3 overexpression cell lines were evaluated by western blot. After 24 h of treatment of 50 μg/ml AVNs, cell viability was measured by MTT assay. g–h HCT116 and DLD1 cells treated with 50 μg/ml AVNs after transfected with GFP or GFP-DDX3 (*n* = 3, mean ± SD). **g** The level of ROS was measured by flow cytometry. h CRC cell apoptosis induced by AVNs was assessed via Annexin V/PI method by using flow cytometry. *P < 0.05, **P < 0.01, ***P < 0.001. P-values were calculated by a Student’s *t*-test. All experiments have been replicated three independent times

Immunofluorescence staining indicated that DDX3 is primarily localized to the mitochondria in CRC cells, and AVNs treatment led to decrease both DDX3 expression and its mitochondrial localization (Fig. 5a). Furthermore, cell fractionation analyses confirmed that DDX3 protein expression and mitochondrial localization were attenuated after AVNs treatment (Fig. 5b). In contrast, qPCR assays showed that AVNs treatment had no effect on the mRNA level of DDX3 (Fig. 5c). To investigate whether AVNs affected DDX3 protein stability, the cycloheximide (CHX)-chase assay was conducted to examine DDX3 turnover with or without AVNs treatment. As shown in Fig. 5d, AVNs treatment resulted in the increased degradation of DDX3, indicating that AVNs promoted DDX3 degradation in CRC cells. Next, the proteasome inhibitor MG-132 or the lysosome inhibitor chloroquine was used to determine the degradation pathway of DDX3 caused by AVNs. The results showed that MG-132 but not chloroquine could block AVNs-induced DDX3 degradation (Fig. 5e, left panel). The DDX3 ubiquitination assay further confirmed that AVNs-induced DDX3 degradation was dependent on the ubiquitin–proteasome system (Fig. 5e, right panel). To elucidate the role of DDX3 on AVNs-induced cell apoptosis, DDX3 protein was overexpressed in DLD1 and HCT116 cells (named GFP-DDX3) using lentiviral infection. The results showed that transient transfection of DDX3 obviously promoted cell viability, and the growth inhibition of CRC cells caused by AVNs treatment was rescued by DDX3 overexpression (Fig. 5f). We then wonder whether AVNs-induced ROS production and apoptosis were through targeting DDX3. As shown in Fig. 5g, h, the DDX3 overexpression significantly reversed the effects of AVNs on ROS production and the apoptosis induction. Consistently, the impaired expressions of subunits of complex I (NDUFS2) and III (UQCRC1) by AVNs treatment were also reversed during DDX3 overexpression (Supplementary Fig. S2B). Taken together, these results suggested that AVNs target DDX3 to trigger ROS-associated cellular apoptosis in CRC cell lines.

### **AVN A i**s a potent DDX3-binding compound in AVNs extracts

**Fig. 6:**
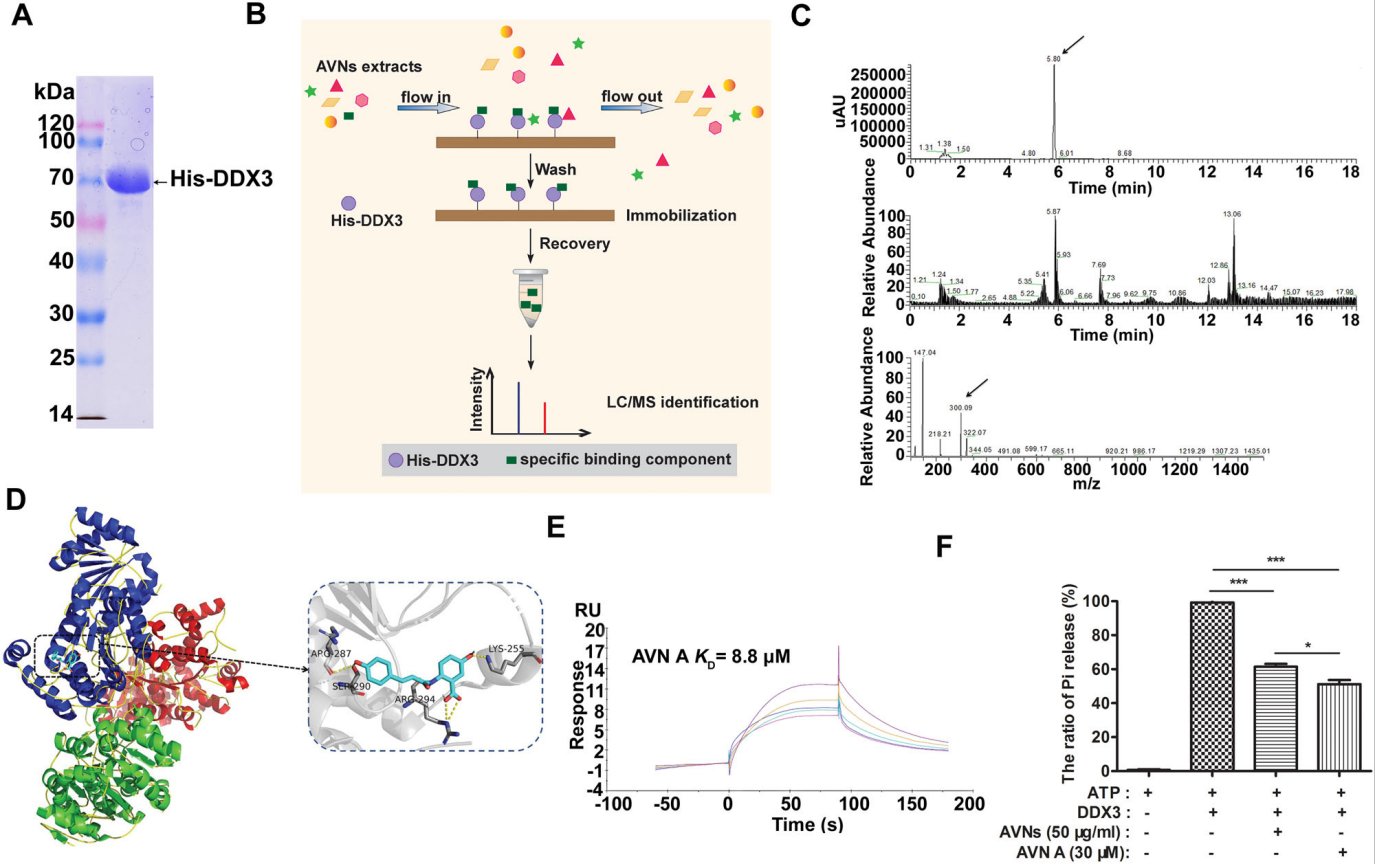
Identification of AVN A as a potent DDX3-bound ingredient from AVNs extracts. **a** SDS-PAGE of His-DDX3 recombinant proteins after purification by nickel affinity column chromatography. **b** Schematic demonstration of the injection and recovery of DDX3-bound ingredients using SPR biosensor. **c** HPLC/MS was carried out to identify DDX3-bound ingredients. The upper is HPLC chromatograms at 280 nm of DDX3-bound ingredients. The lower is the MS spectra of DDX3-bound ingredients. **d** The binding mode of AVN A with DDX3 based on molecular modeling experiments. A detailed view showed that AVN A (cyan) formed hydrogen bond (yellow dotted line) with key amino acid residues. **e** SPR assay was carried out to determine the binding affinity of DDX3 to AVN A. **f** Malachite green assay for ATP hydrolysis showing effects of 50 μg/ml AVNs or 30 μM AVN A on ATPase activity of DDX3. (*n* = 3, mean ± SD). *P < 0.05, **P < 0.01, ***P < 0.001. *P*-values were calculated by a Student’s *t*-test. All experiments have been replicated three independent times

To further identify the functional bioactive compounds that target DDX3 in AVNs extracts, the full length DDX3 was cloned, and His-tagged DDX3 was expressed and purified (Fig. 6a). Figure 6b depicts the schematic overview of the experimental workflow. Bioactive compounds bound to DDX3 immobilized on surface plasmon resonance (SPR) sensor chip could be recovered by Biacore T200 system. The recovered agent from AVNs extracts were detected by HPLC/MS (Fig. 6c) and Nuclear Magnetic Resonance (NMR) spectroscopy (Supplementary Fig. S3A), which was identified as AVN A. To evaluate the binding possibility of AVN A to DDX3, Autodock 6 software suite was used to construct a model of the AVN A-DDX3 complex with the most favorable binding free energies and reasonable orientations, which based on crystal structure of the active catalytic core of DDX3 (PDB ID: 5E7I)^20^. Molecular docking analysis showed that AVN A could form hydrogen bonds with Lys255, Arg287, Ser290, and Arg294 residues in ATP-binding cleft of DDX3 (Fig. 6d). To further validate if AVN A played a critical role in targeting DDX3, SPR affinity assay was performed. Figure 6e showed the association and dissociation binding profile of DDX3 following the addition of different concentrations of AVN A. It indicated that AVN A binds directly to DDX3 with the affinity constant (*K*_*D*_) value of 8.8 μM. MTT assay showed that AVN A significantly suppressed growth of DLD1 but exhibit almost no effects on FHC within a certain dose range (Supplementary Fig. S3B). Malachite green assay was applied to evaluate whether AVNs or AVN A affect ATPase activities of DDX3. The results showed that either AVNs or AVN A could competitively combine DDX3 with ATP and inhibit the ATPase activity of DDX3 (Fig. 6f). These results highlight that AVN A is identified as a novel active compound of AVNs that directly targets the ATP-binding domain of DDX3.

### AVN A directly binds to Arg287 and Arg294 residues of DDX3

**Fig. 7:**
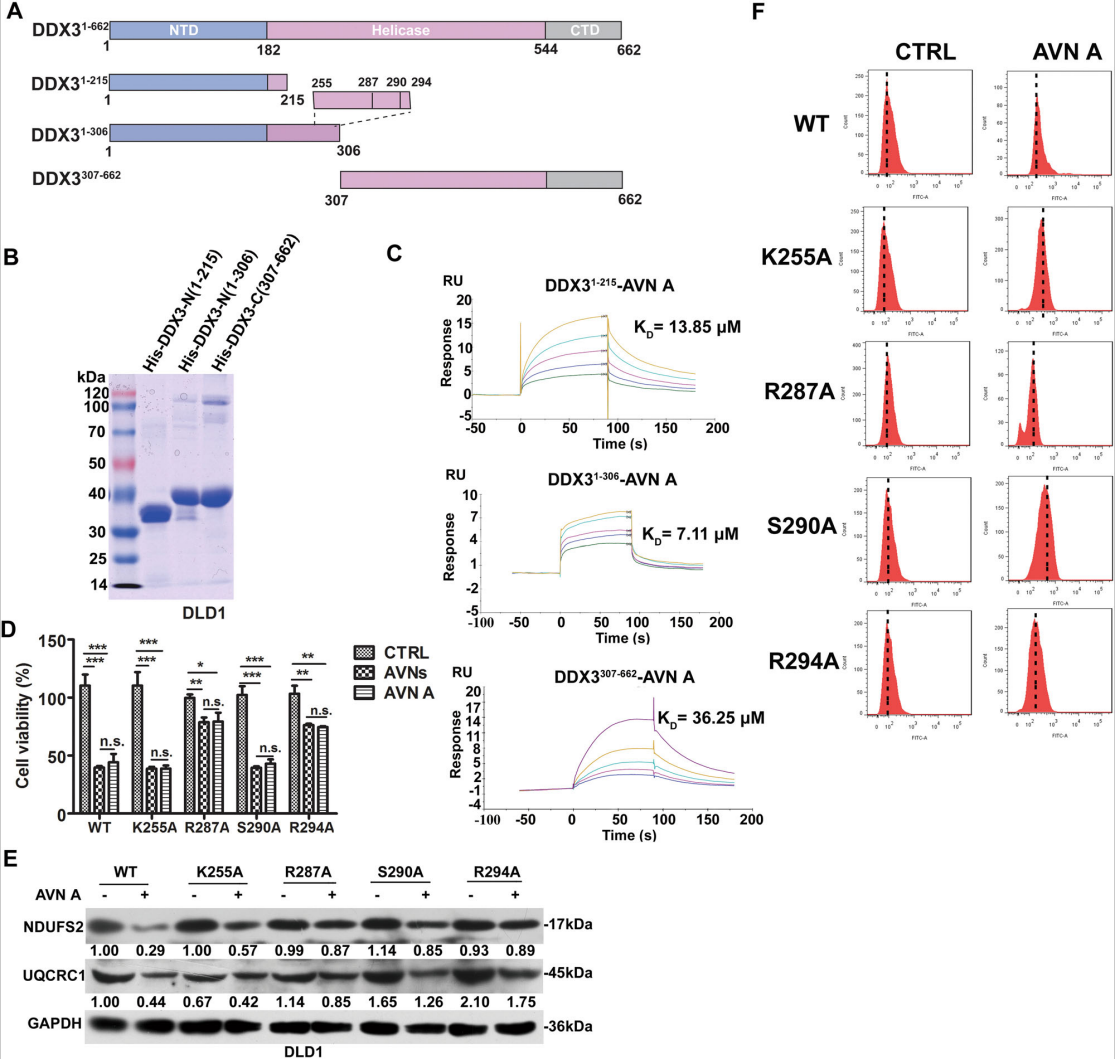
Exploring the binding sites between AVN A and DDX3. **a** Schematic representation of the truncation mutants of human DDX3. NTD, N-terminal regulatory domain; Helicase, Helicase domain; CTD, C-terminal regulatory domain. **b** Three truncation mutants of DDX3 were purified and determined by SDS-PAGE. **c** SPR assay was used to evaluate the binding affinities of three truncation mutants of DDX3 to AVN A. **d** Mutations K255A, R287A, S290A, and R294A in the ATP-binding pocket of DDX3 were performed by site-directed mutagenesis. DLD1 cells were transfected with wild-type DDX3 or the four site mutants, and then treated with 50 μg/ml AVNs or 30 μM AVN A, after 24 h cell viabilities were detected by MTT assay (*n* = 3, mean ± SD). **e** The expression of NDUFS2 and UQCRC1 in wild-type or the 4 DDX3 site mutants were determined by western blot. **f** The levels of ROS in wild-type or the four site mutants of DDX3 treated with 30 μM AVN A were measured by flow cytometry. *P < 0.05, **P < 0.01, ***P < 0.001. *P*-values were calculated by a Student’s *t*-test. All experiments have been replicated three independent times

To further investigate the protein domains that mediate DDX3-AVNs interaction, three mutants DDX3-N1 (aa 1-215), DDX3-N2 (aa 1-306) and DDX3-C1 (aa 307-662) were constructed and expressed in *E. coli* (Fig. 7a, b). Subsequently, SPR-based binding analysis was carried out for in-depth investigation of the interaction between AVN A and DDX3 mutants. As shown in Fig. 7c, AVN A has bound to DDX3-N2 (*K*_*D*_ = 7.11 μM), which is associated with ATP-binding domain, much tighter than DDX3-N1 (*K*_*D*_ = 13.85 μM) and DDX3-C1 (*K*_*D*_ = 36.25 μM) (Fig. 7c). These results were consistent with previous data^21^, and emphasized the ATP-binding domain of DDX3 as a promising target for developing DDX3 inhibitors. In order to clarify which mutant is responsible for anti-tumor property of AVN A, we compared the expression of NDUFS2 and UQCRC1 in DDX3 mutant cell lines (Supplementary Fig. S4A). Our results revealed that DDX3-N2 mutants but not DDX3-N1 and DDX3-C1 abrogated the decreased expression of NDUFS2 and UQCRC1 caused by AVN A (Supplementary Fig. S4B). Based on Fig. 6d, we then construct four site mutants of DDX3 to explore their critical amino acid sites mediating the interaction of DDX3 with AVN A. The lysine, serine and arginine residues in the ATP-binding domain were each replaced with alanine (DDX3-K255A, DDX3-R287A, DDX3-S290A, and DDX3-R294A) by site-directed mutagenesis (Supplementary Fig. S4C). MTT assay showed that the viability of DDX3-R287A and DDX3-R294A mutants treated by AVN A were much higher in contrast to other two mutants in DLD1 cells (Fig. 7d). Western blot analysis further revealed that DDX3-R287A and DDX3-R294A mutants had higher NDUFS2 and UQCRC1 levels after AVN A treatment, compared to the other two mutants (Fig. 7e). Moreover, the ROS production induced by AVN A, was much lower in DDX3-R287A and DDX3-R294A mutants than the other two mutants (Fig. 7f). The protein level of DDX3 was supressed after AVNA treatment (Supplementary Fig. S4D). Taken together, these data demonstrate that AVN A exerts its anti-tumor properties through directly targeting ATP-binding domain of DDX3 at the amino acid residues Arg287 and Arg294.

### AVNs and AVN A inhibit CRC growth by targeting DDX3 in vivo

**Fig. 8:**
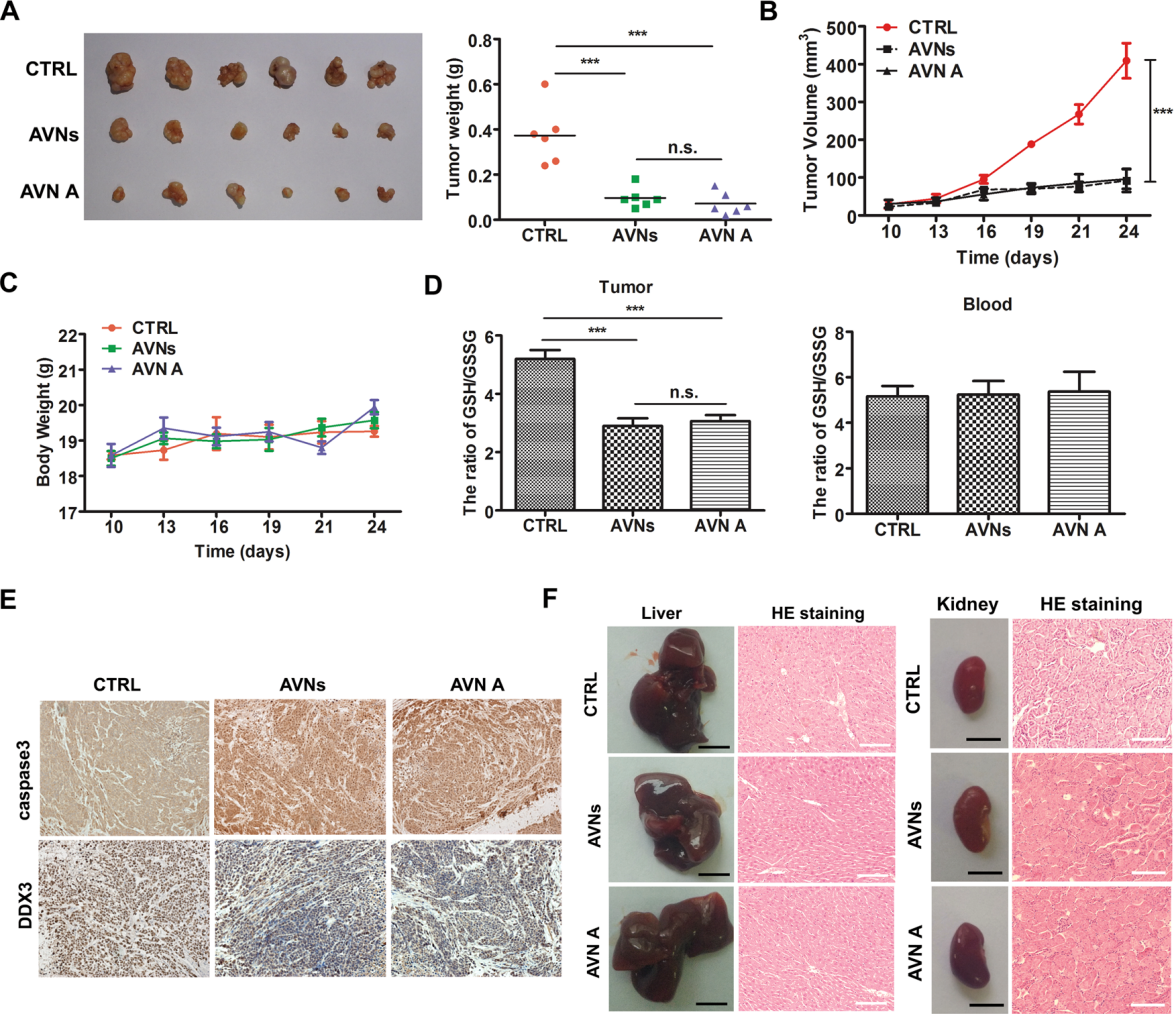
Targeting DDX3 contributes to the in vivo anti-tumor activity of AVNs and AVN A. **a** BALB/c nude mice with DLD1 xenograft treated with vehicle, AVNs and AVN A via oral administration for three weeks. The tumor weight of mice from each group was measured. (*n* = 6, mean ± SD). **b** The tumor volume from each group was monitored. (*n* = 6, mean ± SD). **c** The relative body weight was evaluated during the treatment. (*n* = 6, mean ± SD). **d** The GSH/GSSG ratio in lysates of the tumors tissue (upper) and peripheral blood (lower) in mice administered by AVNs and AVN A were determined. **e** Immunohistochemical stained with antibodies against Caspase3 and DDX3 in tumor sections obtained from all vehicle, AVNs and AVN A treated groups. *P < 0.05, **P < 0.01, ***P < 0.001. P-values were calculated by a Student’s *t*-test. All experiments have been replicated of three independent times. **f** The mice livers and kidneys from the same experiment as (A) were histopathologically evaluated. Scale bars: the black scale bar is 0.5 cm (left) and the white scale bar is 100 mm (right)

A xenograft tumor model was constructed using BALB/c nude mice to assess the in vivo anti-tumor effects of AVNs and AVN A. DLD1 cells inoculation induced tumor formation in nude mice. AVNs (50 mg/kg) and AVN A (30 mg/kg) were orally administered every day for three weeks. Tumor weights in both AVNs and AVN A treatment groups were similar and significantly less than the control group (Fig. 8a). The results shown in Fig. 8b demonstrated that, compared to the vehicle group, the kinetic tumor growth was markedly suppressed in mice treated with AVNs or AVN A (Fig. 8b). There were no obvious distinctions in body weight among the three groups (Fig. 8c). We then examined the effects of AVNs and AVN A on redox state by detecting GSH and GSSG levels in mice tumors. Both AVNs and AVN A treatment dramatically suppressed the GSH/GSSG ratio. In contrast, the GSH/GSSG ratio did not change in blood, suggesting that neither AVNs nor AVN A treatment could cause redox disorder in circulation (Fig. 8d). To further confirm the therapeutic efficacy of AVNs and AVN A in vivo, we examined the expression of Ki67, caspase-3 and DDX3 in mice tumor tissues by IHC staining. The data indicated that AVNs or AVN A significantly suppressed the expression of Ki67 and DDX3 and induced cleaved-caspase-3 expression in the xenograft tumors (Fig. 8e). In addition, neither AVNs nor AVN A treatment caused liver or kidney injury (Fig. 8f). These in vivo results thus suggest that AVNs and AVN A can be employed as anti-tumor active agents for the clinical trials of CRC.

## Discussion

Epidemiological studies have suggested an inverse relationship between long term intake of oats diet and incidence of CRC^3^. Herein, we found that AVNs extracts were effective against CRC cells and significantly reduced the tumor size without drastic side effects. Accumulating evidence suggest that solid tumors reside in abnormal microenvironments, such as nutrient depletion, hypoxia, and low extracellular pH, which constitute a pathological barrier to the delivery of therapeutic agents to tumors^14^. Accumulating studies reported that hypoxia and low extracellular pH attenuate therapeutic efficacy and are mainly contributors to the poor outcome of chemotherapeutics, due to the acquisition of chemoresistance by tumor cells in these hostile conditions^22,23^. Notably, our study has demonstrated that tumor cells are more vulnerable to AVNs treatment under these stress milieus (Fig. 1c), which further revealed AVNs had good penetration characteristics and maintain inhibitory efficacy against CRC cells. The present study thus provides a novel strategy to break the pathological barriers, which may help overcome chemoresistance in clinical trials.

It is well documented that tumor cells characteristically require more ATPs to confront with cellular stress responses or harsh microenvironment, which is closely related to mitochondria^24^. A growing body of evidence suggest that targeting mitochondria is a promising therapeutic strategy against tumors^25^. Herein, our results demonstrated that AVNs targets mitochondria that results in the mitochondrial swelling and bioenergetics imbalance (Fig. 2a–c, e). AVNs significantly impaired ETC complexes, as demonstrated by decreased expression of ETC complexes subunits such as ND2, ND5, CYTB, COX2, and ATP6. The impaired ETC complexes further results in the oxidative stress and excessive ROS generation to surpass a fatal threshold, with ensuing PTP activation, MMP loss, cytochrome C release, culminating in apoptosis of CRC cells.

How does AVNs affect mitochondrial ETC complexes? We found that AVNs exhibited anti-tumor activities by targeting DDX3, an ATPase-dependent RNA helicase, which is implicated in transcriptional regulation, mRNA splicing, mRNA export and mitochondrial translation^26^. Recently, Heerma et al. reported that DDX3 is a key regulator of mitochondrial translation and its inhibitor RK-33 enhanced the sensitivity of radiotherapy in breast cancer via blocking mitochondrial translation^18^. In the present work, we found that DDX3 was overexpressed in 53% (28/53) CRC samples and the downregulation of ETC complex I and III subunits induced by AVNs were reversed by DDX3 overexpression. Consistent with previous literature regarding the DDX3 roles in mitochondrial translation^18^, the reduction in mitochondrial localization of DDX3 by AVNs treatment intuitively supports that AVNs can suppress mitochondrial translation. Thus, the most likely scenario is that AVNs target DDX3 and suppress DDX3 expression, resulting in impaired translation of ETC complexes components and ROS-mediated bioenergetics catastrophe and apoptosis.

Our results demonstrated that AVNs significantly reduces the GSH/GSSG ratio, which creates a more oxidative intracellular microenvironment in CRC cells. However, several prior studies have highlighted that AVNs possess antioxidant activities^27^. What is the reason for the contradiction between our findings and others? The key to solve the puzzle relies on DDX3. Firstly, DDX3 is highly expressed in CRC cells as compared to normal cells. Secondly, the basal level of ROS is low in normal cells but is much higher in CRC cells. Thus, AVNs can target DDX3 and trigger excessive production of ROS in CRC cells. Therefore, AVNs exhibit pro-oxidant effects and trigger ROS-mediated selective apoptosis in CRC cells. In contrast, due to the low basal ROS level and trace amount of DDX3, the ROS in normal cells can be neutralized by the antioxidant activity of AVNs. In this way, AVNs treatment can protect normal cells from oxidative stress. This opinion is consistent with the available literature that several phytochemicals showed both pro-oxidant and antioxidant properties to exert their anti-cancer effects^28^. We also present a good explanation why AVNs could selectively kill CRC cells but not normal cells. Considering the properties such as to selectively kill cancer cells and stronger anti-tumor capacity under stress conditions, AVNs are indeed promising candidates for CRC therapy^29^.

Through the combination of SPR and HPLC/MS molecular docking, we identified AVN A as a novel and effective DDX3 ligand. Another intriguing question aroused in the present study is that how AVNs affect DDX3 function? We uncovered that AVN A binds to the Arg287 and Arg294 residues in the ATP-binding domain, decreased ATPase activities and induced degradation of DDX3.

In conclusion, our work demonstrates that AVN A, the bioactive agent of AVNs, exhibits anti-cancer activities with low toxicity in vitro and in vivo. AVN A directly binds to the ATP-binding domain of oncogenic protein DDX3, selectively kills tumor cells in which DDX3 are highly expressed. Therefore, AVN A can be considered as a novel potential candidate for CRC treatment, and it is worthy of further research and investigations.

## Materials and methods

### Chemicals and antibodies

Oats bran was provided by Chinese Academy of Agricultural Sciences. AVN A (purity > 99%) was purchased from Topharman (shanghai, China). CHX, MTT and NAC were obtained from Sigma (St. Louis, MO). MG-132 and Chloroquine diphosphate were purchased from TargetMol (Shanghai, China). ROS probe DCFH-DA staining, Mitochondrial membrane potential assay kit with JC-1, GSH and GSSG Assay Kit were purchased from Beyotime Biotech (Beijing, China). The Annexin V-FITC Apoptosis Detection kit was obtained from KeyGEN BioTECH (Nanjing, China). MitoTracker Red was obtained from Invitrogen (Carlsbad, CA). EdU assay kit was from Ribobio (Guangzhou, China). The antibodies against NDUFS2, UQCRC1, ND2, ND5, CYTB, COX2, ATP6, and ATP5A1 were purchased from Proteintech (Wuhan, China). Caspase-3, Ubiquitin, DDX3 and GFP antibodies were obtained from Cell Signaling Technology (Beverly, MA). Tim 23 and Cytochrome C antibodies were from Abmart (Shanghai, China). GAPDH antibody was purchased from Affinity Biosciences (Cincinnati, USA). Malachite Green Phosphate Assay Kit was purchased from Cayman (MI, USA).

### Extraction and isolation of avenanthramides

Oats bran was provided by Chinese Academy of Agricultural Sciences and extracted as previously described with minor modification^5^. Briefly, oats bran was ground to fine powder and extracted with solvents including alcohol, deionized water, acetic acid (800: 199:1) at 80 °C for 2 h with three cycles. After removed lipid, the extracts were centrifuged at 11,000 × *g* for 10 min at room temperature. The supernatant was used for decompress filtration and the residue was re-extracted. The AVNs extracts were obtained by rotary evaporator and stored at −20 °C before use.

### HPLC/MS analysis of AVNs

AVNs extracts from oats bran were analysed by HPLC/MS using a Thermo Scientific Q Exactive Orbitrap mass spectrometer equipped with an electrospray ionization (ESI) source according to previous reports^30^.

### Cell culture, cell proliferation and clonogenic assay

Human colon cancer cell lines HCT116, DLD1, SW480, SW620, HT29, and Human colon normal epithelium FHC were purchased from the ATCC (Manassas, VA). The detailed methods of cell culture were described in our previous studies^31^. Cell proliferation was detected by MTT assay and EdU assay^32^. A clonogenic assay was carried out as previously reported^32^.

### Metabolic flux analysis

Seahorse XF glycolysis stress test kit and seahorse XF cell mito stress test kit (Seahorse Bioscience, USA) were used to determine continuously the oxygen consumption rates (OCR) and extracellular acidification rate (ECAR) by Seahorse XF24 instruments as previously described^33^.

### ROS dectection, mitochondrial membrane potential, and GSH and GSSG assay

Intracellular ROS level was determined by the fluorescence probe DCFH-DA (Beyotime, Beijing, China) as per manufacturer’s recommendations. A mitochondrial membrane potential assay kit (Beyotime, China) was used to measure mitochondrial membrane potential (MMP). Effect of AVNs on the ratio of GSH/GSSH was measured by a commercially available GSH and GSSG Assay Kit (Beyotime, China).

### Animal experiment, histology and immunohistochemistry (IHC)

DLD1 cells (1 × 10^6^ suspended in 100 μL PBS) were subcutaneously injected into the right flank of 4–5 week-old female nude mice. When the tumors were palpable, mice were randomly divided into three groups (*n* = 6). One group received standard drinking water (control group) and the other two groups were orally administered with AVNs (50 mg/kg) and AVN A (30 mg/kg) separately in the drinking water every day till the completion of study. After sacrificing, tumor masses were collected, weighted, frozen in liquid nitrogen and subsequently used for biochemical and immunohistochemistry analysis (IHC). For IHC assay, sections were incubated with specific antibodies against Caspase 3 and DDX3 overnight at 4 °C. Samples were stained with haematoxylin–eosin (HE) to indicate nucleus and cytoplasm, respectively. All protocols involving live mice were approved by the Animal Care and Use Committee of Shanxi University.

### Tissue specimens

A total of 53 pairs of clinical sample sections of the colon tumors and adjoining normal tissue were obtained from the First Hospital Affiliated with Shanxi Medical University according to the Institutional Research Board approved protocol. All sections were stained with DDX3 for IHC assay. The current study was approved by the Ethics Committee of Shanxi Medical University.

### Plasmid constructs, expression and purification of DDX3 and DDX3 mutants

The cDNA encoding human DDX3 was cloned into pLVX-AcGFP1-N1 and pET28a for cell transfection and expression, respectively. The different truncated DDX3 mutants were amplified from the cDNA of human DLD1 cells and then cloned into pLVX-AcGFP1-N1, named pLVX-AcGFP1-K255A, -R287A, -S290A, and R294A were generated by the Easy Mutagenesis System of Transgen Biotech (Beijing, China) and verified by DNA sequence analysis.

### Recognition and recovery of DDX3-bound ingredients and SPR affinity analysis

A biacore T200 system (GE Healthcare, Sweden) was applied for SPR analysis and recognition and recovery of DDX3-bound ingredients according to literature^34^. SPR response curves and the dissociation constant at equilibrium (*K*_*D*_) were evaluated and analyzed by Biacore evaluation software.

### Autodock, ATPase Activity assay, and Transmission electron microscopy

All molecular docking calculations were performed using Autodock Vina program as previously described^21^. Malachite green assay was performed to measure the production of inorganic phosphate during ATP hydrolysis by DDX3 as previously described^35^. Transmission electron microscopy was used to observe the effects of AVNs treatment on mitochondrial morphology in HCT116 and DLD1 cells as described previously^36^.

### Statistical analysis

Statistical analyses were performed using the Student’s two-tailed *t*-tests and Mann-Whitney U-test. All analyses were performed using GraphPad Prism Software (San Diego, CA, USA). *p*-values <0.05 were considered as statistically significant.

## Supplementary information

supplement

Figure S1

Figure S2

Figure S3

Figure S4

Figure S5

Figure S6

Figure S7

## References

[1] Brenner H, Kloor M, Pox CP (2014). Colorectal cancer. Lancet.

[2] Shanmugam MK (2016). Cancer prevention and therapy through the modulation of transcription factors by bioactive natural compounds. Semin. Cancer Biol..

[3] Yu X, Yang M, Dong J, Shen R (2018). Comparative analysis of the antioxidant capacities and phenolic compounds of oat and buckwheat vinegars during production processes. J. Food Sci..

[4] Meydani M (2009). Potential health benefits of avenanthramides of oats. Nutr. Rev..

[5] Ishihara A, Kojima K, Fujita T, Yamamoto Y, Nakajima H (2014). New series of avenanthramides in oat seed. Biosci. Biotechnol. Biochem..

[6] Scarpa ES, Antonini E, Palma F, Mari M, Ninfali P (2018). Antiproliferative activity of vitexin-2-O-xyloside and avenanthramides on CaCo-2 and HepG2 cancer cells occurs through apoptosis induction and reduction of pro-survival mechanisms. Eur. J. Nutr..

[7] Bhola PD, Letai A (2016). Mitochondria-judges and executioners of cell death sentences. Mol. Cell.

[8] Sabharwal SS, Schumacker PT (2014). Mitochondrial ROS in cancer: initiators, amplifiers or an Achilles' heel?. Nat. Rev. Cancer.

[9] Bol GM, Xie M, Raman V (2015). DDX3, a potential target for cancer treatment. Mol. Cancer.

[10] Wu DW (2016). DDX3 enhances oncogenic KRASinduced tumor invasion in colorectal cancer via the betacatenin/ZEB1 axis. Oncotarget.

[11] He TY (2016). DDX3 promotes tumor invasion in colorectal cancer via the CK1epsilon/Dvl2 axis. Sci. Rep..

[12] Wu DW, Lin PL, Wang L, Huang CC, Lee H (2017). The YAP1/SIX2 axis is required for DDX3-mediated tumor aggressiveness and cetuximab resistance in KRAS-wild-type colorectal cancer. Theranostics.

[13] Hitayezu R, Jason Kinnin MMB, Henderson K, Tsopmo A (2018). Antioxidant activity, avenanthramide and phenolic acid contents of oat milling fractions. J. Cereal Sci..

[14] Dehne N, Mora J, Namgaladze D, Weigert A, Brune B (2017). Cancer cell and macrophage cross-talk in the tumor microenvironment. Curr. Opin. Pharmacol..

[15] Plitzko B, Kaweesa EN, Loesgen S (2017). The natural product mensacarcin induces mitochondrial toxicity and apoptosis in melanoma cells. J. Biol. Chem..

[16] Shrotriya S (2015). Grape seed extract targets mitochondrial electron transport chain complex III and induces oxidative and metabolic stress leading to cytoprotective autophagy and apoptotic death in human head and neck cancer cells. Mol. Carcinog..

[17] Stevens JF, Revel JS, Maier CS (2017). Mitochondria-centric review of polyphenol bioactivity in cancer models. Antioxid. Redox Signal..

[18] Heerma van Voss MR, Vesuna F (2018). Targeting mitochondrial translation by inhibiting DDX3: a novel radiosensitization strategy for cancer treatment. Oncogene.

[19] Wilky BA (2016). RNA helicase DDX3: a novel therapeutic target in Ewing sarcoma. Oncogene.

[20] Zhang L (2017). Discovery of a small molecule targeting ULK1-modulated cell death of triple negative breast cancer in vitro and in vivo. Chem. Sci..

[21] Bol GM (2015). Targeting DDX3 with a small molecule inhibitor for lung cancer therapy. EMBO Mol. Med..

[22] Klemm F, Joyce JA (2015). Microenvironmental regulation of therapeutic response in cancer. Trends Cell Biol..

[23] Taylor S (2015). Microenvironment acidity as a major determinant of tumor chemoresistance: Proton pump inhibitors (PPIs) as a novel therapeutic approach. Drug Resist Updat..

[24] Nakazawa MS, Keith B, Simon MC (2016). Oxygen availability and metabolic adaptations. Nat. Rev. Cancer.

[25] Weinberg SE, Chandel NS (2015). Targeting mitochondria metabolism for cancer therapy. Nat. Chem. Biol..

[26] Ariumi Y (2014). Multiple functions of DDX3 RNA helicase in gene regulation, tumorigenesis, and viral infection. Front Genet..

[27] Fu J (2015). Oat avenanthramides induce heme oxygenase-1 expression via Nrf2-mediated signaling in HK-2 cells. Mol. Nutr. Food Res..

[28] Chikara S (2018). Oxidative stress and dietary phytochemicals: Role in cancer chemoprevention and treatment. Cancer lett..

[29] Hussain SS, Kumar AP, Ghosh R (2016). Food-based natural products for cancer management: is the whole greater than the sum of the parts?. Semin. Cancer Biol..

[30] Cui L, Liu J, Yan X, Hu S (2017). Identification of metabolite biomarkers for gout using capillary ion chromatography with mass spectrometry. Anal. Chem..

[31] Yang P, Li Z, Fu R, Wu H (2014). Pyruvate kinase M2 facilitates colon cancer cell migration via the modulation of STAT3 signalling. Cell Signal..

[32] Yang P (2018). Tannic acid directly targets pyruvate kinase isoenzyme M2 to attenuate colon cancer cell proliferation. Food Funct..

[33] Huang R (2017). Monomethyltransferase SETD8 regulates breast cancer metabolism via stabilizing hypoxia-inducible factor 1α. Cancer lett..

[34] Chen L (2018). Biosensor-based active ingredients recognition system for screening STAT3 ligands from medical herbs. Anal. Chem..

[35] Samal SK, Routray S, Veeramachaneni GK, Dash R, Botlagunta M (2015). Ketorolac salt is a newly discovered DDX3 inhibitor to treat oral cancer. Sci. Rep..

[36] Ding GB (2018). Robust anticancer efficacy of a biologically synthesized tumor acidity-responsive and autophagy-inducing functional Beclin 1. Acs. Appl. Mater. Interfaces.

