# Supplementary material for: Avenanthramide A triggers potent ROS-mediated anti-tumor effects in colorectal cancer by directly targeting DDX3
Source: Cell Death Dis. 2019 Aug 7;10(8):593. doi: 10.1038/s41419-019-1825-5 (PMC6685981; doi:10.1038/s41419-019-1825-5)
Supplement: Supplementary file 1 — supplement [file 41419_2019_1825_MOESM1_ESM.docx]

**Supplemental Figure and Figure Legends**

**Figure S1. AVNs inhibits viability of CRC cells.**

(A) CRC cells (DLD1, SW480, SW620 and HCT116) and normal colonic epithelial cells (FHC) were treated with 10 μg/ml AVNs for 24 h, 48 h or 72 h, and cell viability was detected using MTT. (B) Effects of AVNs on the growth of DLD1 and HCT116 cells were tested by the clonogenic survival assay. (C) Colony formation ratio was determined by dissolved in acetic acid and detected in 570 nm. (D) Density plot analysis of ROS level in FHC cells with AVNs treatment was measured by flow cytometry.

**Figure S2. DDX3 is a target of AVNs in CRC cells.**

(A) The protein levels of DDX3 under the stress milieus (combination of nutrient depletion, hypoxia, and low extracellular pH) in HCT116 and DLD1 cells were detected by Western blot. (B) HCT116 and DLD1 cells treated with 50 μg/ml AVNs after transfected with GFP or GFP-DDX3 (n = 3, mean ± SD). The expression of NDUFS2 and UQCRC1 were determined by Western blot.

**Figure S3. Inhibitory effects of AVN A on the proliferation of CRC cells.**

(A) NMR spectroscopy was applied for the identification of the recovered agent from AVNs extracts. (B) CRC cells DLD1 and normal colonic epithelial cells FHC were treated with different concentrations of AVN A for 24 h, and cell viability was detected using MTT.

**Figure S4. Effects of AVN A on truncation mutants of GFP-DDX3 in CRC cells.**

(A) To generate cell lines expressing truncation mutants of DDX3, DLD1 cells were transfected with constructs expressing GFP, truncation mutants of GFP-DDX3 and GFP-DDX3, and then analyzed by Western blot using GFP antibody. (B) DLD1 cells were transfected with constructs expressing GFP-DDX3 and truncation mutants of GFP-DDX3 for 24 h and then treated with 30 μM AVN A for 24 h. The expression of NDUFS2 and UQCRC1 were determined by western blot. (C) The identification of DDX3 point mutant recombinant plasmids by sequencing. (D) HCT116 and DLD1 cells treated with 30 μM AVN A and the expression of DDX3 was determined by western blot.

**Figure S5.** Uncropped western blots for data shown in Fig. 3A, 3F and 3G.

**Figure S6.** Uncropped western blots for data shown in Fig. 4C and 4D.

**Figure S7.** Uncropped western blots for data shown in Fig. 5B, 5D, 5E, 7E, S2A, S2B, S4B and S4D.
