# Supplementary figures and images for: Avenanthramide A triggers potent ROS-mediated anti-tumor effects in colorectal cancer by directly targeting DDX3
Source: Cell Death Dis. 2019 Aug 7;10(8):593. doi: 10.1038/s41419-019-1825-5 (PMC6685981; doi:10.1038/s41419-019-1825-5)

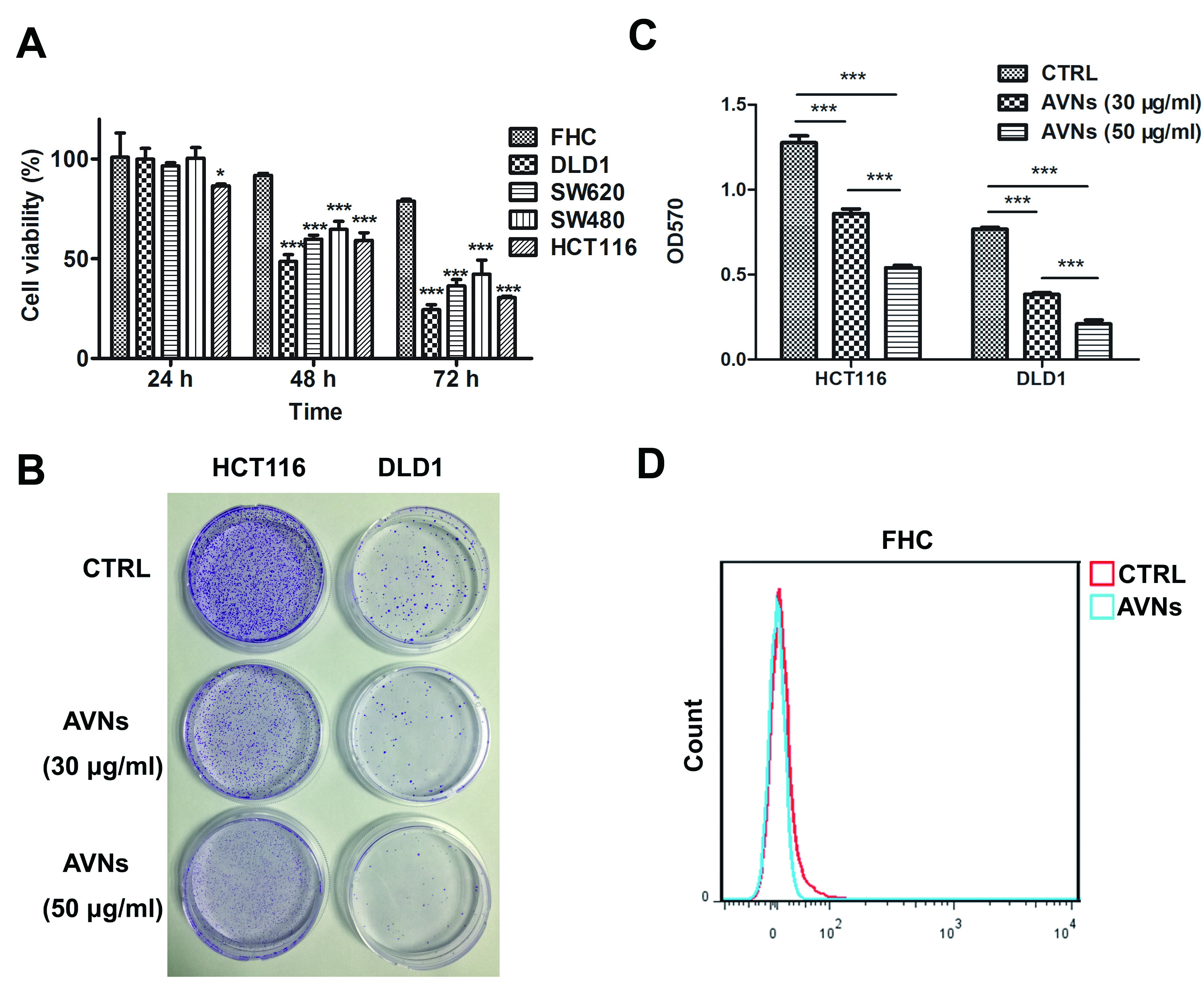

Supplement: Supplementary file 2 — Figure S1 [file 41419_2019_1825_MOESM2_ESM.jpg]

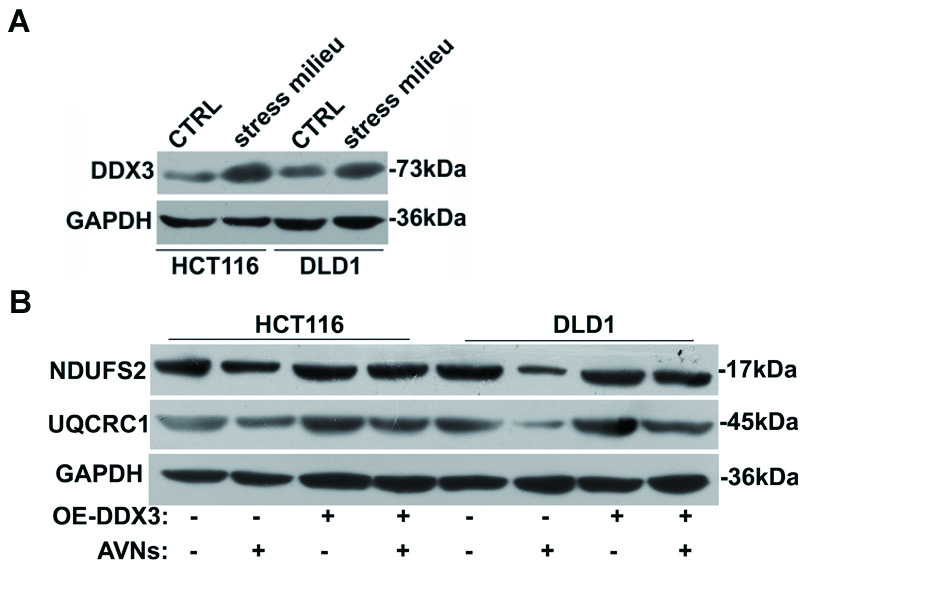

Supplement: Supplementary file 3 — Figure S2 [file 41419_2019_1825_MOESM3_ESM.jpg]

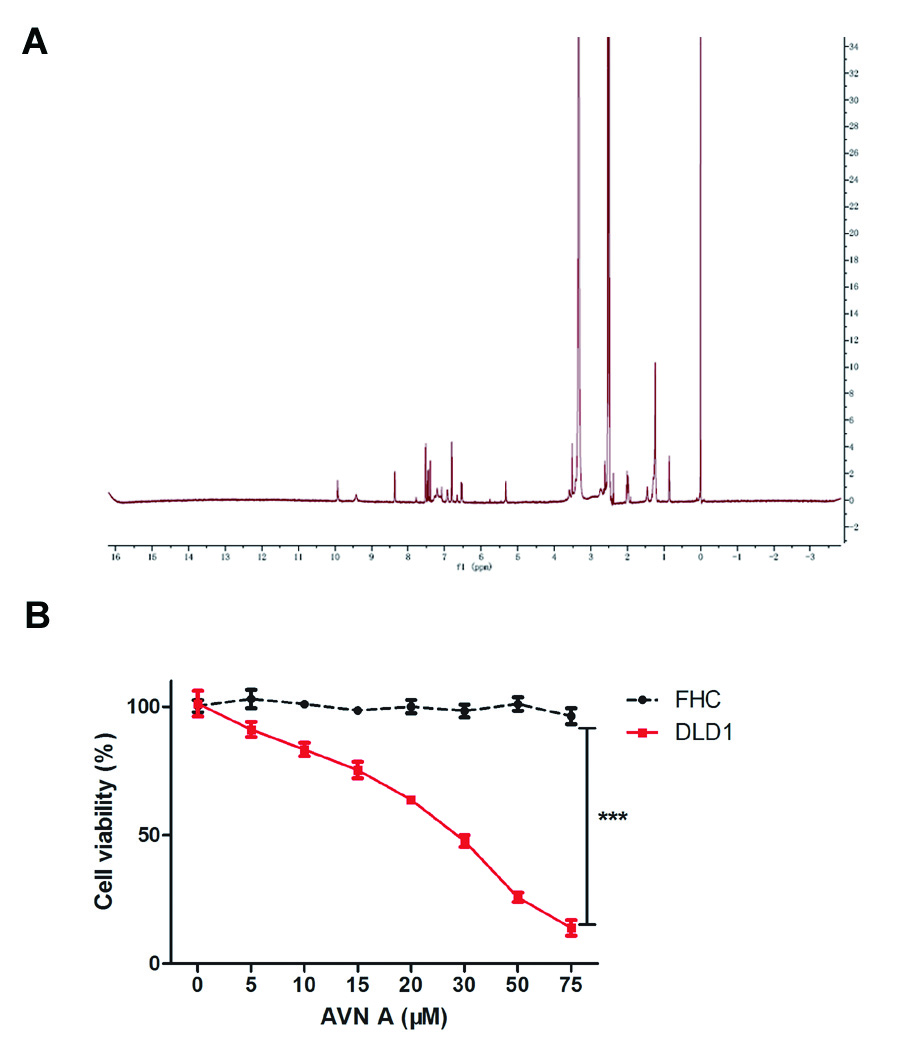

Supplement: Supplementary file 4 — Figure S3 [file 41419_2019_1825_MOESM4_ESM.jpg]

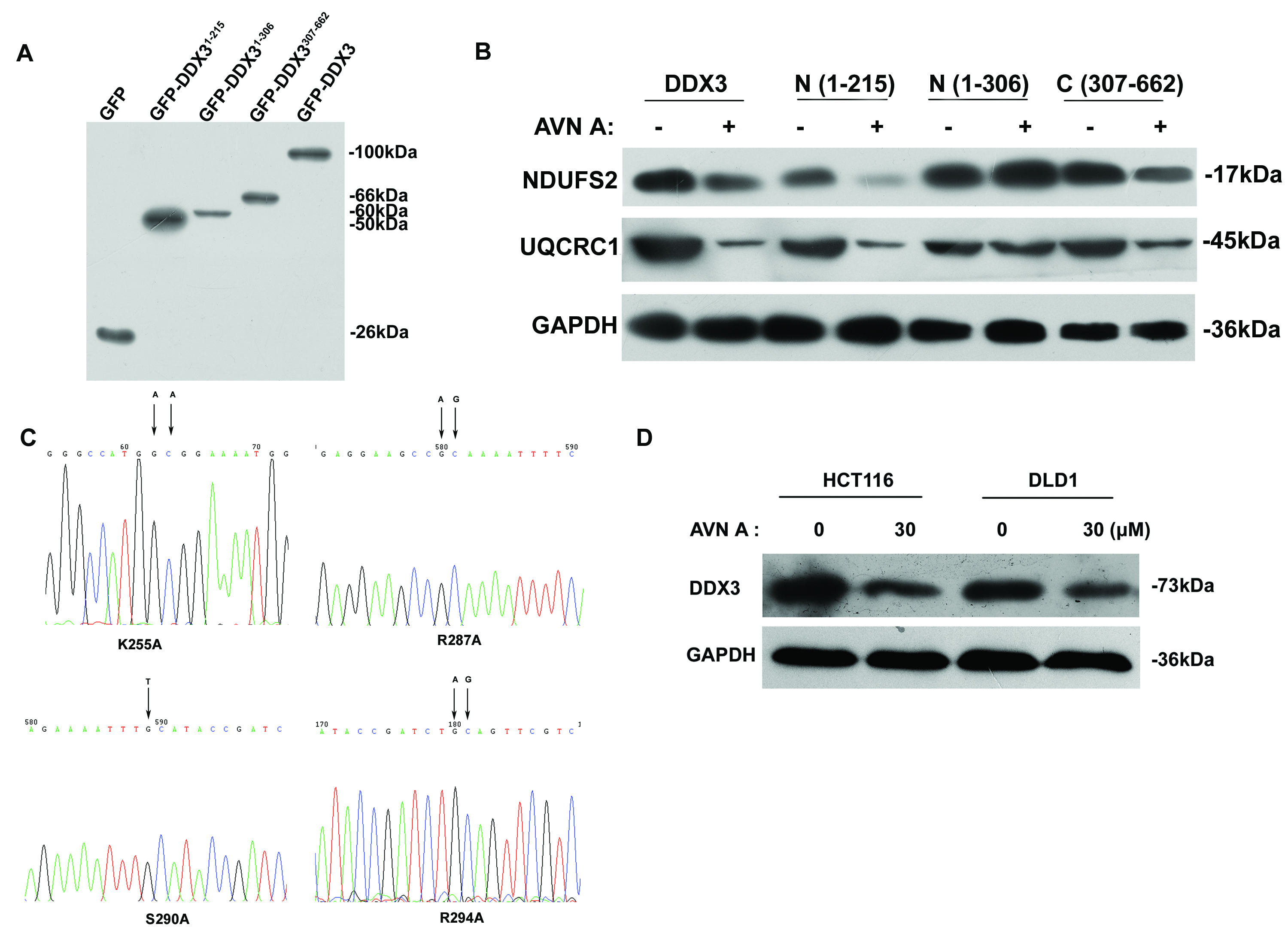

Supplement: Supplementary file 5 — Figure S4 [file 41419_2019_1825_MOESM5_ESM.jpg]

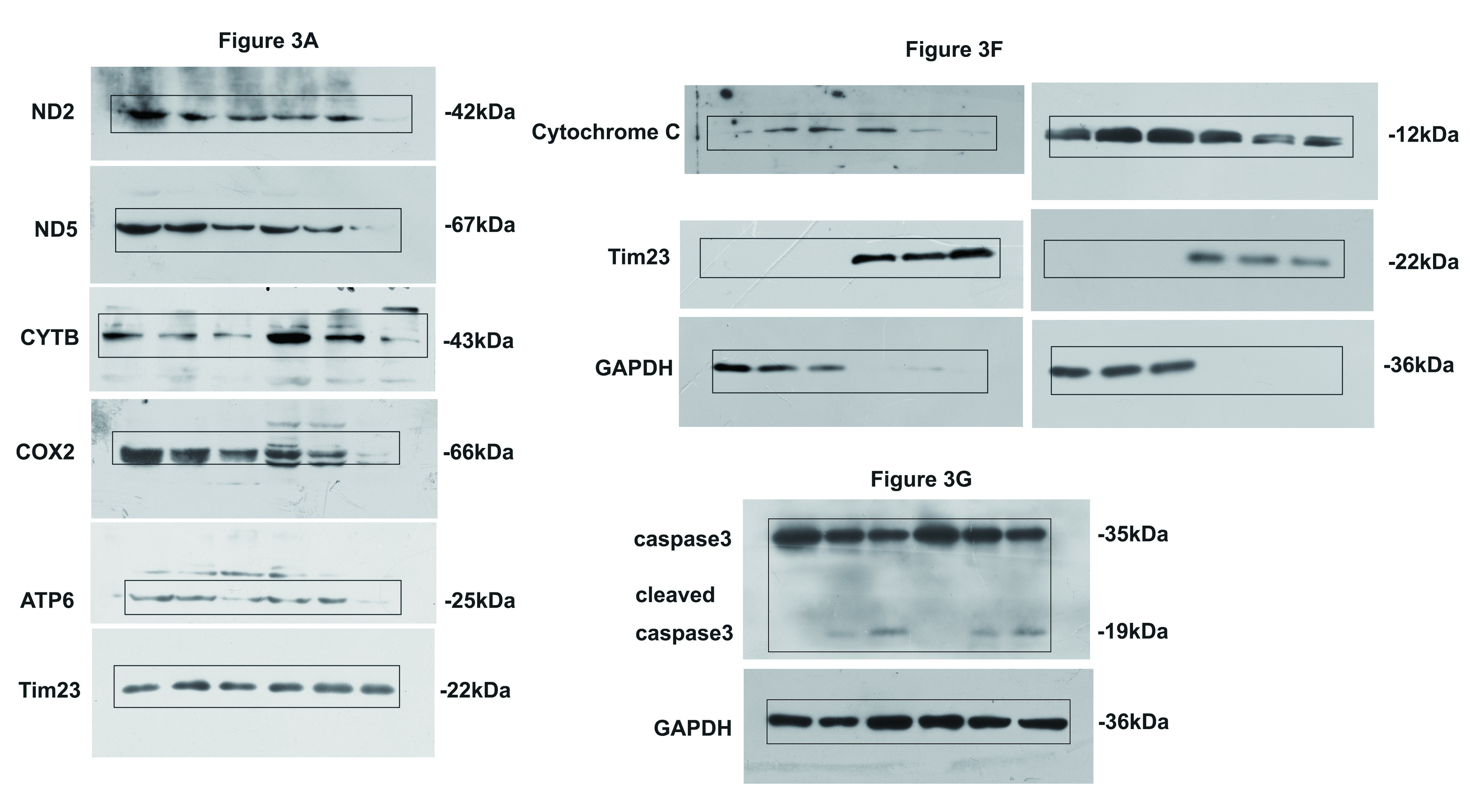

Supplement: Supplementary file 6 — Figure S5 [file 41419_2019_1825_MOESM6_ESM.jpg]

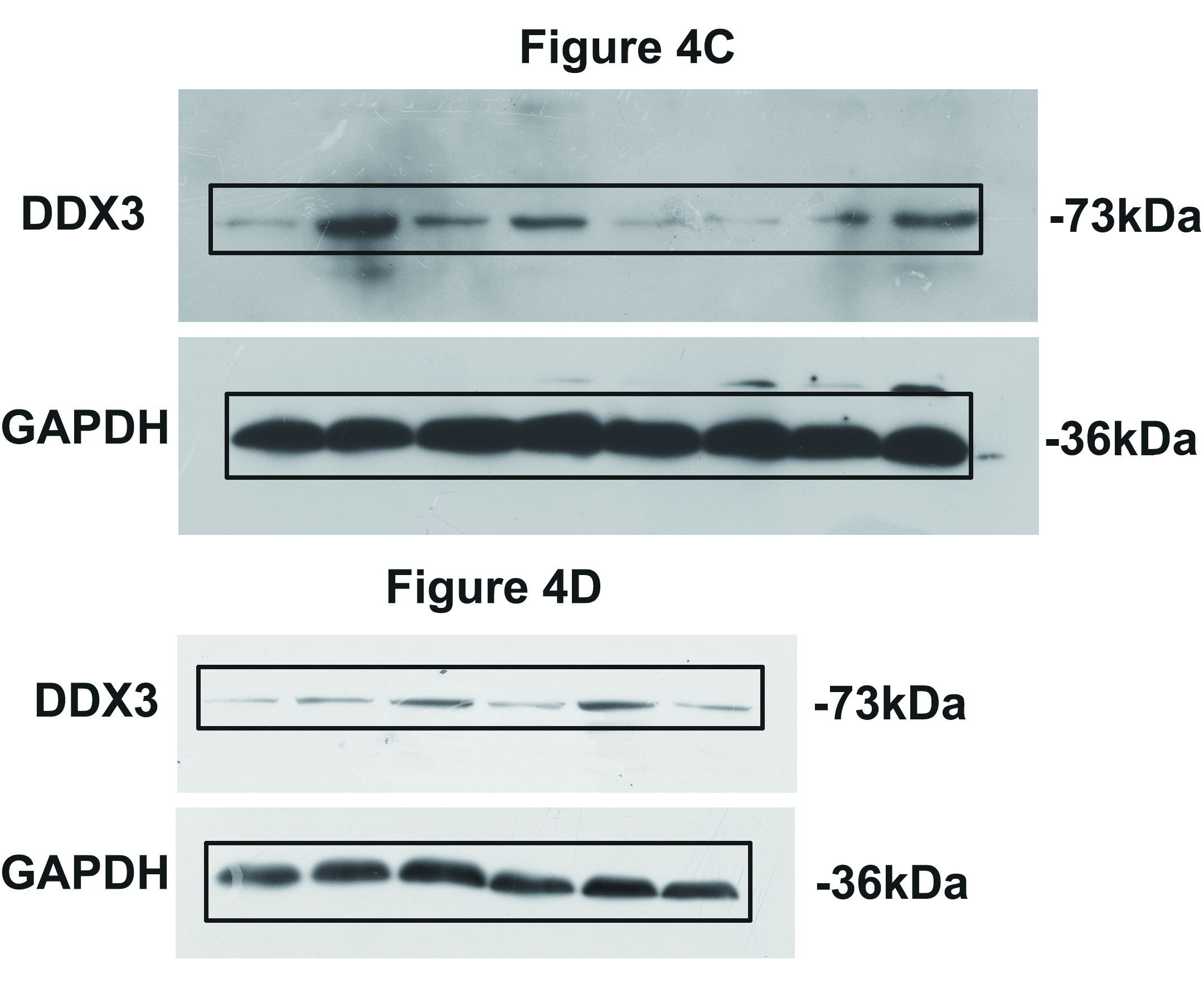

Supplement: Supplementary file 7 — Figure S6 [file 41419_2019_1825_MOESM7_ESM.jpg]

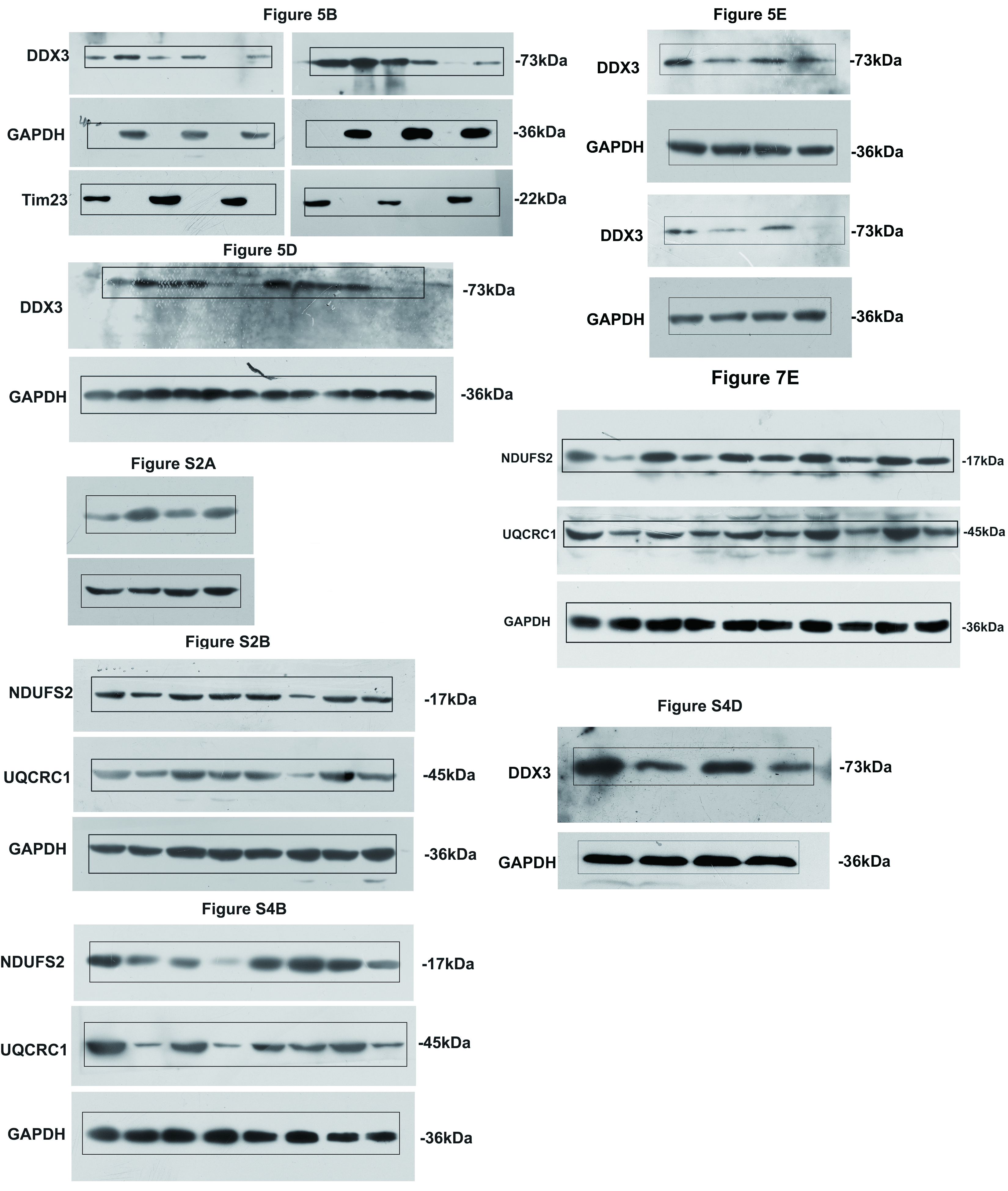

Supplement: Supplementary file 8 — Figure S7 [file 41419_2019_1825_MOESM8_ESM.jpg]
